# Influence of socioeconomic factors on female entrepreneurship: an analysis using structural equation modeling (PLS-SEM)

**DOI:** 10.3389/fsoc.2025.1684697

**Published:** 2025-10-17

**Authors:** Rosa Ysabel Bazán Valque, Omer Cruz Caro, William Humberto Portilla Bazán, Elías Alberto Torres Armas, Cesar Jefferson Samillan Vasquez, Carlos Andres Rojas Puerta

**Affiliations:** Universidad Nacional Toribio Rodríguez de Mendoza de Amazonas, Chachapoyas, Peru

**Keywords:** gender equality, women-led businesses, access to productive resources, entrepreneurial skills, social empowerment

## Abstract

Female entrepreneurship has emerged as a strategy for reducing poverty, generating employment opportunities, and promoting gender equality, particularly in rural areas where structural inequalities persist. However, there is still a limited understanding of how socioeconomic factors influence their development. This study aimed to analyze the influence of these factors on women-led entrepreneurship, considering dimensions such as family income, social empowerment, economic growth, access to resources, entrepreneurial capabilities, and sociocultural factors. The research was conducted with a sample of 98 female entrepreneurs from the province of Chachapoyas, Amazonas region of Peru, providing context-specific evidence for understanding female entrepreneurship in a rural Amazonian setting rarely studied. The initial least squares structural equation modeling (PLS-SEM) technique was employed, supported by SmartPLS4 software. The results indicate that socioeconomic factors have a positive impact on income, empowerment, and economic growth. Furthermore, female entrepreneurship strengthens capacities, facilitates access to productive resources, and contributes to transforming restrictive cultural norms. Beyond the financial benefits, it acts as a driver of social change. This study offers a comprehensive framework for understanding female entrepreneurship. It provides useful evidence for the design of public policies promoting women's economic inclusion in historically excluded areas.

## 1 Introduction

Unemployment and poverty are serious problems affecting all countries, and their impact has intensified in recent years due to the health crisis and the financial recession, which caused a rapid increase (Zhang et al., 2025). In response to this, several projects and strategies have been implemented worldwide to reduce these rates, among which the business efforts of the countries stand out particularly (Bierwiaczonek and Pyka, 2023; Roy et al., 2022). As a response to this, entrepreneurship stands out, which involves the process of planning, establishing, and managing a new commercial enterprise intended to generate profits, while considering financial risks and possible losses (Zhang et al., 2025). Therefore, it is considered an engine of innovation, economic growth, employment, and improved living standards (Morante et al., 2024; Porfírio et al., 2024). However, countries often face certain challenges in promoting successful entrepreneurship, including access to credit, market dynamics, and regulatory barriers (Agarwal et al., 2022). In this sense, women play an essential role in business activities and are likely to bring about significant change (Bierwiaczonek and Pyka, 2023). With their exceptional capabilities and skills, they contribute to innovation, creativity, productivity, and overall economic growth (Borisov and Vinogradov, 2022; Xheneti et al., 2019).

A significant transformation, emphasized by both sustainable innovation and sustainable entrepreneurship, and highlighted in the 2030 Agenda, is the greater inclusion of women in all business sectors (Alexeeva-Alexeev et al., 2025). SDG 5, Gender Equality, specifically calls for empowering all women and girls to reduce gender disparities. A key target of this goal, “Ensure women's full and effective participation and equal opportunities for leadership at all levels of decision-making in political, economic, and public life,” underscores the need to foster female entrepreneurship and promote women to senior and middle management positions.

According to the latest report, Global Entrepreneurship Monitor (GEM) (2024a), the rate of female startups globally increased by four percentage points between 2021 and 2023 compared to two decades ago. The proportion of established businesses owned by women has also increased, albeit by just over one and a half percentage points. However, in advanced economies, female entrepreneurship is declining (Alexeeva-Alexeev et al., 2025). Despite improvements in women's perceptions of business opportunities and their entrepreneurial skills, their fear of failure has increased significantly. In terms of female leadership in management roles, the latest report by Marks et al. (2014) indicates that there are now more women in senior management positions worldwide than 3 or 5 years ago. However, these gains remain concentrated in specific industries, particularly the service sector, and regions such as North America, Europe, and Asia-Pacific. Despite these advances, the overall number of women in management positions remains low. Barriers to promotion and appointment to leadership positions can stem from motherhood, difficulty balancing work and personal life, and a lack of mentors (Hurley and Choudhary, 2016). In addition, the attitudes of male executives, a lack of female role models, and a lack of confidence among women can further hinder career advancement (De Mascia, 2015).

The Report Global Entrepreneurship Monitor (GEM) (2024b) reveals that women continue to be significantly involved in high-potential ventures across the 45 countries surveyed in 2023. One in three high-growth entrepreneurs and nearly two in five export-oriented startups were led by women. Countries such as China, Colombia, Iran, Lithuania, the Netherlands, and Venezuela showed particularly high rates of women bringing innovations to market. Meanwhile, women were one-fifth less likely to report exiting a business than men, on average, with the highest rates in low-income countries and the lowest in middle-income countries. Perceptions of entrepreneurship among women have improved significantly over the past two decades, with a 79% increase in the perception of business opportunities and a 27% increase in entrepreneurial skills. Fear of failure has also increased by more than half among women, raising new questions for researchers and policymakers. Ownership rates for businesses founded by women in the 30 countries compared have also increased from 4.2% to the current 5.9%. High rates were especially high for women in South Korea, Saudi Arabia, Lithuania, Puerto Rico, and Thailand.

Female entrepreneurship has gained momentum due to its potential to drive economic growth, innovation, and social progress (Chatterjee et al., 2022; Dheer et al., 2019). However, female entrepreneurship rates vary widely across countries (Deng et al., 2024). Necessity-based female entrepreneurship is driven by a lack of viable employment opportunities, while opportunity-based female entrepreneurship arises from aspirations for economic independence and personal fulfillment (Dencker et al., 2021). Typically, necessity-based female entrepreneurship thrives in developing countries, while opportunity-based female entrepreneurship thrives in developed countries (Coffman and Sunny, 2021). However, some developing countries, such as Malaysia, Thailand, Turkey, and Vietnam, have higher rates of opportunity-based rather than necessity-based female entrepreneurship, which is consistent with trends in developed countries in Europe and the Americas (Global Entrepreneurship Monitor (GEM), 2019).

Female entrepreneurship in Peru has spanned various productive sectors, with beauty being the most prominent, accounting for 74.5% of these businesses. Retail trade ranks second with 61.9%, followed by food and beverage service activities at 59.9% and wholesale trade at 57.8% (Ramos, 2023). Between 2019 and 2024, the number of women entrepreneurs increased by 1%, on average. In 2024, the number rose to around 2.3 million women, slightly exceeding pre-pandemic levels (2019). Furthermore, in 2024 alone, 42.8% of startups in the country were led by women, reflecting their significant participation in the entrepreneurial ecosystem. Despite this, significant challenges remain, including a lack of access to financing and the predominance of informality (Observatorio PRODUCEmpresarial., 2025).

Female entrepreneurs often face a series of structural obstacles that limit their development in the entrepreneurial field. These include difficulties in accessing financing due to limited access to credit and financial institutions that favor male entrepreneurs (Avşar and Avşar, 2021; Kaur and Kaur, 2024). Added to this is the lack of formal education and business training, which restricts their ability to manage and sustain businesses efficiently (Agrawal et al., 2023; Ladan and Abubakar, 2024). In addition, gender discrimination and traditional roles continue to discourage female participation in entrepreneurial activities (Bianco et al., 2017; Mistry, 2024), while the scarce presence of public policies specifically directed at women aggravates these inequalities (Agrawal et al., 2023; Kaur and Kaur, 2024). Despite these barriers, it is recognized that female entrepreneurship can significantly contribute to reducing gender gaps, as many women manage to overcome and resist these structural challenges (Bianco et al., 2017). However, the persistence of these problems highlights the need for more comprehensive and sustained strategies that foster an inclusive business ecosystem.

The gender gap in the entrepreneurial ecosystem is exacerbated by social, economic, and cultural factors (Hechavarría and Ingram, 2019; Lizunova and Mindruta, 2022; Ozkazanc-Pan and Clark Muntean, 2021). Therefore, the importance of supporting ecosystems for women's business success is highlighted, especially in crisis contexts (Samsami et al., 2024). Family and social network support have a significant effect on reducing these gaps, particularly in impoverished areas (Welsh et al., 2023). Likewise, it has been shown that the existence of favorable conditions within the ecosystem enhances female participation in entrepreneurship (Simmons et al., 2019), while countries with higher levels of gender equality tend to have smaller disparities in this area (Rietveld and Patel, 2022). Factors such as economic participation and political empowerment are also crucial to closing the gap (Halabisky and Shymanski, 2023). However, women continue to face obstacles even after business failure, due to social stigma and fear of failure (Marineau et al., 2022).

It is in this context that the study aims to analyze the influence of socioeconomic factors on female entrepreneurship. To this end, a partial least squares structural equation model (PLS-SEM) was developed to explore how variables such as household income, economic growth, social empowerment, access to resources, entrepreneurial capabilities, and sociocultural factors influence the development of female-led ventures. The purpose is to provide evidence that will inform the design of policies and programs that promote gender equality in the entrepreneurial ecosystem, from a comprehensive perspective and with a real impact in the most vulnerable contexts.

This study is presented in such a way that it begins with the introduction, which contextualizes the problem of female entrepreneurship and highlights its social and economic relevance. It then presents the theoretical framework that conceptually underpins the research and serves as the basis for the development of the model and the formulation of the hypotheses, in which the proposed variables and relationships are specified. Next, it describes the methodology used for data collection and analysis, and then presents the results obtained through structural equation modeling. Afterwards, it discusses the main findings together with their implications and limitations, and finally, it presents the conclusions and recommendations derived from the study.

## 2 Theoretical framework

Female entrepreneurship refers to the participation of women in “self-employment”, where they are small business owners, but do not create a new company (Deng et al., 2021)and to “entrepreneurship” in the classic sense, where women take the initiative, mobilize resources, and take risks to create a new business (Jennings and Brush, 2013). The decision to become an entrepreneur can be driven by “push” or “pull” factors (Deng et al., 2024; Jennings and Brush, 2013). Furthermore, since women tend to focus more on social goals than men, this type of entrepreneurship can generate greater benefits for their families and communities (Minniti, 2010). For example, when faced with poverty, women entrepreneurs spend more of their earnings on feeding, clothing, and educating their children, while men spend more on clothing, entertainment (including alcohol), and food for themselves (Downing, 1991; Nichter and Goldmark, 2009). However, despite the importance of female entrepreneurship in these contexts, it remains under-researched and under-theorized (Amine and Staub, 2009; Shahriar and Shepherd, 2019). In particular, we know little about the wellbeing of these women entrepreneurs (Chatterjee et al., 2022).

Female entrepreneurship is considered from a gender perspective, emphasizing the need to understand how social norms and institutional barriers affect women entrepreneurs (Muntean and Ozkazanc-Pan, 2015). Traditional comparisons between male and female entrepreneurs do not take into account the specific challenges that women face, such as access to financing and networks (Seckin-Halac and Samur-Teraman, 2022). Female entrepreneurship serves as a mechanism for social mobility, enabling women to improve their economic situation and contribute to gender equality (Moiseeva, 2024). The expansion of opportunities for women in business is linked to broader economic growth and societal progress, highlighting the transformative potential of female entrepreneurship (Agarwal and Agrawal, 2023).

The term “woman entrepreneur” covers a wide range of roles, from self-employed women to those running larger businesses, reflecting the diversity of women's entrepreneurial experiences (Hasanova, 2022). This broad definition underscores the importance of recognizing the diverse forms of entrepreneurship and the different contexts in which women operate (McAdam, 2022). Although attention to female entrepreneurship often focuses on empowerment and social mobility, it is essential to consider the persistent structural inequalities that continue to hinder women's full participation in the business world (De Souza, 2024). Addressing these barriers remains crucial to achieving true gender equality in entrepreneurship (Lingappa et al., 2023).

Women entrepreneurs represent the fastest-growing category of entrepreneurship worldwide and have received, especially in recent years, the attention of many academics (Cardella et al., 2020). According to emerging literature, women can make a significant contribution to entrepreneurial activity (Noguera et al., 2013) and economic development (Hechavarria et al., 2019; Kelley et al., 2017) in terms of job creation and increased gross domestic product (GDP) (Ayogu and Agu, 2015; Bahmani-Oskooee et al., 2013), with positive impacts on reducing poverty and social exclusion (Langowitz and Minniti, 2007; Rae, 2014). The percentage of women who decide to pursue a business career is, however, lower than that of men (Elam et al., 2019), and this difference is greater as the level of development of the country increases (Coduras and Autio, 2013).

Despite the widely reported obstacles that women face in male-dominated societies, the proportion of women entering entrepreneurship in the developing world has increased markedly in recent decades (Bullough et al., 2022; Gatewood et al., 2009; Yousuf and Lawton, 2012). It is worth noting that women entrepreneurs have become key players in economic development through their entrepreneurial activity (Brush et al., 2019; Bruton et al., 2011; Hechavarria et al., 2019). Access to financial resources for women entrepreneurs indeed contributes to some extent to combating discrimination and, more importantly, increases their access to equity capital and loans (Henry et al., 2017), especially in developing countries (Simba et al., 2023). With this level of empowerment, female entrepreneurship can be an engine that drives economic and social development (Hechavarria et al., 2019), not only for women but also for the economies of many parts of developing countries (Adom, 2015; Nziku and Henry, 2021).

Female entrepreneurship is important for people, communities, and countries (Elam et al., 2019; Minniti and Naudé, 2010). Research recognizes that female entrepreneurship contributes to the stability and wellbeing of communities and provides economic opportunities to disadvantaged groups, including women, low-income individuals, and minorities (Ascher, 2012; Kairiza et al., 2017). Although a much larger number of women in the developing world are reported to be illiterate and live in poor communities (Bruton et al., 2021; Ojong, 2019), female entrepreneurship allows them to participate in local economies, and the process helps them become entrepreneurs (Elam et al., 2019; Frešer et al., 2019).

Studies argue that a lack of resources repeatedly limits women entrepreneurs due to the complex barriers they face (Hahn et al., 2020; Xu et al., 2020). This implies that family integration is crucial for female entrepreneurs, since the family environment provides access to resources, especially when family members provide support (Zhang et al., 2025). Therefore, studies assess how various factors, in the presence of family support, could be decisive for business success, focusing on women, since support for a specific gender could be affected by their social role in the family sphere (Dewitt et al., 2023; Shastri et al., 2022).

Furthermore, they show that a significant proportion of female entrepreneurship operates informally, which has important implications for motivations and sustainability strategies. For example, in Peru, informality accounts for about 53% of gross domestic product due to unregulated activities, many of which are led by women microentrepreneurs who have difficulty accessing formal financing and institutional networks (Silupu et al., 2024). In Latin America and the Caribbean, around 21.2% of businesses started by women are done so out of necessity, suggesting that many do not have access to formal opportunities or structured support to undertake more sustainable ventures (Correa, 2024). At the same time, research on informal female entrepreneurship has documented that women use family networks, informal credit, and diversification of activities as key strategies to sustain their businesses in the face of formal barriers such as registration, taxes, or legal protection (Silupu et al., 2024; Xheneti and Madden, 2025).

In informal settings, women's motivations tend more toward necessity than opportunity. Economic constraints, lack of formal employment, and labor market precarity push many women to start businesses for subsistence rather than innovation. In Latin America, studies show that necessity-driven entrepreneurship predominates in rural and peri-urban areas, where structural barriers to formal employment are greatest (Ruiz et al., 2021). Moreover, World Bank surveys in Latin America and the Caribbean reveal that household obligations and domestic work reduce women's participation in formal business training programs, reinforcing the idea that informality is often not a voluntary choice but a response to constrained options (Fontes and Bustamante, 2022).

To sustain their informal businesses, women often employ adaptive strategies: they draw on family or community networks for financing; combine multiple income-generating activities; use informal credit; operate without formal registration to avoid regulatory costs; and continuously adapt in the face of economic shocks. Also, informal or non-formal education and training (practical skills, experience-based learning, community-based workshops) are found to enhance the operational resilience of women-led informal enterprises. For example, Essien and Adelekan (2021) demonstrate that informal entrepreneurial education improves women's management skills and is positively linked with business sustainability. Socioeconomic factors significantly influence female entrepreneurship, where economic necessity, lack of access to education and financial resources, and cultural norms often act as barriers, especially in developing economies (Chikh-Amnache and Mekhzoumi, 2024).

Based on the analysis of this study, which addresses how economic resources and knowledge influence female entrepreneurship, we seek to provide empirical evidence that allows for a more comprehensive understanding of the conditions that favor or limit women's participation in entrepreneurial activities, especially in contexts where small businesses predominate. This research aims to close knowledge gaps regarding how these socioeconomic dimensions impact the sustainability and development of female entrepreneurship, and how, in turn, entrepreneurship can become a mechanism for empowerment and social transformation. Consequently, the following research question is posed: How do socioeconomic factors, particularly economic resources and entrepreneurial capacities, influence the development of female entrepreneurship in the province of Chachapoyas?

## 3 Model development and hypothesis formulation

Female entrepreneurship has established itself as a fundamental strategy for promoting gender equality, reducing poverty, and promoting sustainable economic development (Brush et al., 2019; Chatterjee et al., 2022). However, its consolidation does not depend exclusively on personal motivation or the desire for economic independence (Dencker et al., 2021) but it is deeply conditioned by a series of socioeconomic and structural factors (Hechavarría and Ingram, 2019; Pantaleón et al., 2023). In many developing contexts, women's ventures are highly informal, with limited legal registration, restricted access to formal credit, and scarce institutional protection. This reality shapes their motivations, more frequently necessity-driven than opportunity-driven, and the sustainability strategies they employ (Essien and Adelekan, 2021; Global Entrepreneurship Monitor (GEM), 2024a; Ruiz et al., 2021).

Indeed, various studies have shown that unequal access to financial resources (Henry et al., 2017), the lack of support networks (Dewitt et al., 2023), restrictive sociocultural norms (Bullough et al., 2022), limitations in education (Agrawal, 2018), and business training negatively affect women's ability to undertake (Agarwal et al., 2022; Agrawal et al., 2023; Bianco et al., 2017; Marín et al., 2024). In informal settings, women rely heavily on family networks, informal credit, and diversification of activities to sustain their ventures, compensating for formal barriers such as registration, taxes, or legal protection (Essien and Adelekan, 2021).

Based on this evidence, this study proposes a theoretical model based on structural equation modeling (PLS-SEM) that explores the causal relationships between socioeconomic factors and female entrepreneurship. This model considers family income, economic growth, social empowerment, access to resources, entrepreneurial capabilities, and sociocultural factors as latent variables, all of which are influenced by the socioeconomic factors of the environment (Hair et al., 2022; Hechavarria et al., 2019).

It is assumed that socioeconomic factors, understood as the set of structural conditions that affect the quality of life, such as educational level, access to basic services, employment, and financial stability, have a significant impact on family income (H1), economic growth (H2), and social empowerment (H3) of women (Deng et al., 2024; Minniti and Naudé, 2010). Likewise, it is considered that female entrepreneurship directly influences access to resources (H4) and the strengthening of entrepreneurial capacities (H5), since it allows the development of skills, competencies, and key social networks to sustain a business (Cardella et al., 2020; Langowitz and Minniti, 2007). Furthermore, it is suggested that entrepreneurship generates changes in the sociocultural factors of the environment, transforming traditional gender roles and promoting new forms of economic participation (H6) (Bianco et al., 2017; Bullough et al., 2022).

**Figure 1:**
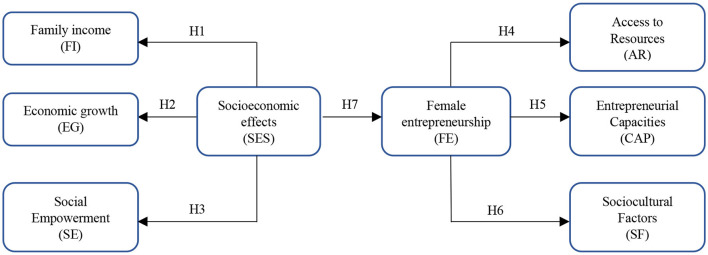
Study framework. *H* = *Hypothesis*.

On the other hand, it is suggested that socioeconomic factors also have a direct effect on the decision to undertake (H7), since a favorable structural and institutional environment allows for reducing entry barriers and provides greater opportunities to start a business, while highly informal settings may constrain these opportunities (Acs et al., 2015; Brixiová et al., 2020). Figure 1 describes the theoretical model of the study and the corresponding hypotheses.

H1: Socioeconomic factors (SES) influence family income (FI).

H2: Socioeconomic factors (SES) influence economic growth (EG).

H3: Socioeconomic factors (SES) influence social empowerment (SE).

H4: Female entrepreneurship (FE) influences access to resources (AR).

H5: Female entrepreneurship (FE) influences entrepreneurial capabilities (CAP).

H6: Female entrepreneurship (FE) influences sociocultural factors (SF).

H7: Socioeconomic factors (SES) influence female entrepreneurship (FE).

## 4 Materials and methods

### 4.1 Place of study

**Figure 2:**
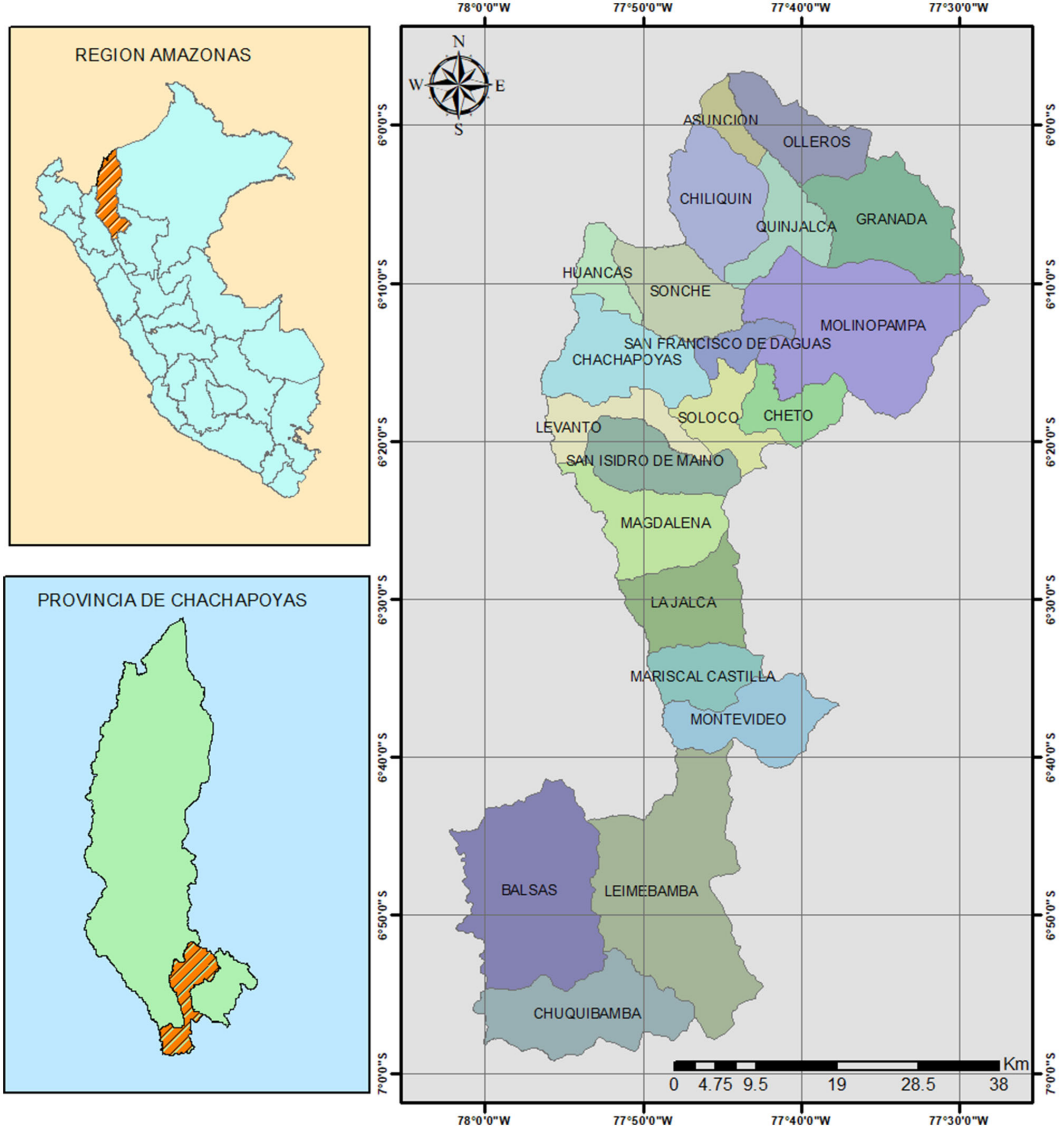
Location of the study.

The study population was women's entrepreneurship in the province of Chachapoyas, Amazonas. Chachapoyas province is located in northeastern Peru and comprises a total of 21 districts (see Figure 2).

### 4.2 Methodology

**Figure 3:**
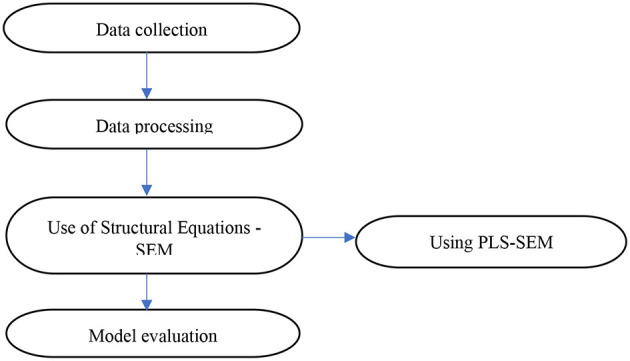
Study methodology.

The study focused on an analysis of the socioeconomic factors associated with female entrepreneurship. A quantitative approach was used through a non-experimental cross-sectional design. The collected data were analyzed using descriptive statistics and structural equation modeling to measure the influence of variables (see Figure 3).

#### 4.2.1 Data collection

The survey technique used was a face-to-face survey, administered in the entrepreneurs' workspaces, such as workshops, stores, or local fairs, and in some cases through home visits. This approach created a sense of trust and ensured participants understood the items. This approach allowed for greater interaction between the interviewer and the respondent, facilitating immediate clarification of questions and ensuring higher-quality data collection.

The research sample was non-probabilistic for convenience, given that we needed women entrepreneurs with a functioning business, and there are no reference data at the institutional level to statistically calculate a study sample, 98 entrepreneurs were registered, and their participation was determined according to the criteria:

- Inclusion criteria: Female entrepreneur over 18 years of age, with a functioning business, and who is under her charge.
- Exclusion criteria: Female entrepreneur under 18 years of age who works in a business but is not in charge.

This strategy ensured that the participants reflected the target population for the research questions and produced high-quality, directly relevant data. Although convenience sampling can limit external generalizability, in exploratory studies using partial least squares structural equation modeling (PLS-SEM), sample sizes of this magnitude are widely accepted and provide robust estimates when reliability and validity requirements are satisfied, following the “10-times rule” and minimum R^2^ guidelines (Hair et al., 2017; Sarstedt et al., 2017). Thus, the present sample offers a solid and credible basis for analyzing the relationships among the variables studied in this population segment.

A self-constructed questionnaire validated by three experts with experience in research related to entrepreneurship was used. The instrument was structured into three main sections: (i) socio-demographic data and moderating variables, (ii) socioeconomic factors, with dimensions of family income, economic growth, and social empowerment, and (iii) female entrepreneurship, with dimensions of access to resources, entrepreneurial capabilities, and sociocultural factors. The instrument included a total of 36 items measured on a 5-point Likert scale (1 = never, 2 = rarely, 3 = sometimes, 4 = almost always, and 5 = always). To ensure its reliability, the questionnaire was validated using Cronbach's alpha reliability test, yielding a coefficient of 0.884 for the socioeconomic factor's questionnaire and a coefficient of 0.921 for female entrepreneurship. In addition, Table 1 provides a concise overview of the operational definitions of the eight constructs analyzed in the study, specifying how each was measured and the theoretical foundations supporting their operationalization.

**Table 1:** Operational definitions of the eight constructs and their theoretical foundations.

**Construct**	**Operational definition**	**Theoretical origin**
Family Income (FI)	The frequency with which the income generated by the business contributes to improving the household economy, covering basic expenses, and enabling savings/investment.	Deng et al., 2024; Minniti and Naudé, 2010
Economic Growth (EG)	The extent to which the business creates jobs, diversifies the local economy, spawns new enterprises, or contributes to the regional GDP.	Brush et al., 2019; Pantaleón et al., 2023
Social Empowerment (SE)	Participation in household and community decision-making, leadership roles, and perception of financial independence and control over one's life.	Langowitz and Minniti, 2007; Bianco et al., 2017
Access to Resources (AR)	Access to financing, family financial support, funding programs, support networks, and business advice.	Henry et al., 2017; Dewitt et al., 2023
Entrepreneurial Capabilities (CAP)	Educational level, use of academic knowledge, prior experience, and learning new skills to manage the business.	Cardella et al., 2020
Sociocultural Factors (SF)	Perception of cultural barriers, gender discrimination, adaptation to cultural norms, and family support for entrepreneurship.	Bianco et al., 2017; Bullough et al., 2022
Female Entrepreneurship (FE)	The degree of direct involvement of the woman in the management and decision-making of her business.	Jennings and Brush, 2013; Agarwal et al., 2022
Socioeconomic Effects (SES)	A composite latent variable reflecting the structural context (education, services, employment, financial stability, and networks) in which the entrepreneur operates.	J. F. Hair et al., 2022; Hechavarría and Ingram, 2019

The questionnaire was administered in person between February and June of the current year at the workplaces and meeting venues of the participating female entrepreneurs. All participants gave their informed consent before the instrument's administration. The study's objectives, the voluntary nature of their participation, and the confidentiality of the data collected were clearly explained to them. They were assured that their responses would be used solely for academic and research purposes and that their identities would not be revealed under any circumstances. This procedure was carried out according to the ethical principles established in the Declaration of Helsinki (World Medical Association, 2013), and by the ethical guidelines in social research that promote respect, autonomy, and protection of participants (Babbie, 2020).

#### 4.2.2 Data processing

The data were processed using descriptive statistics and SEM structural equations, using PLS-SEM 4 software.

**Figure 4:**
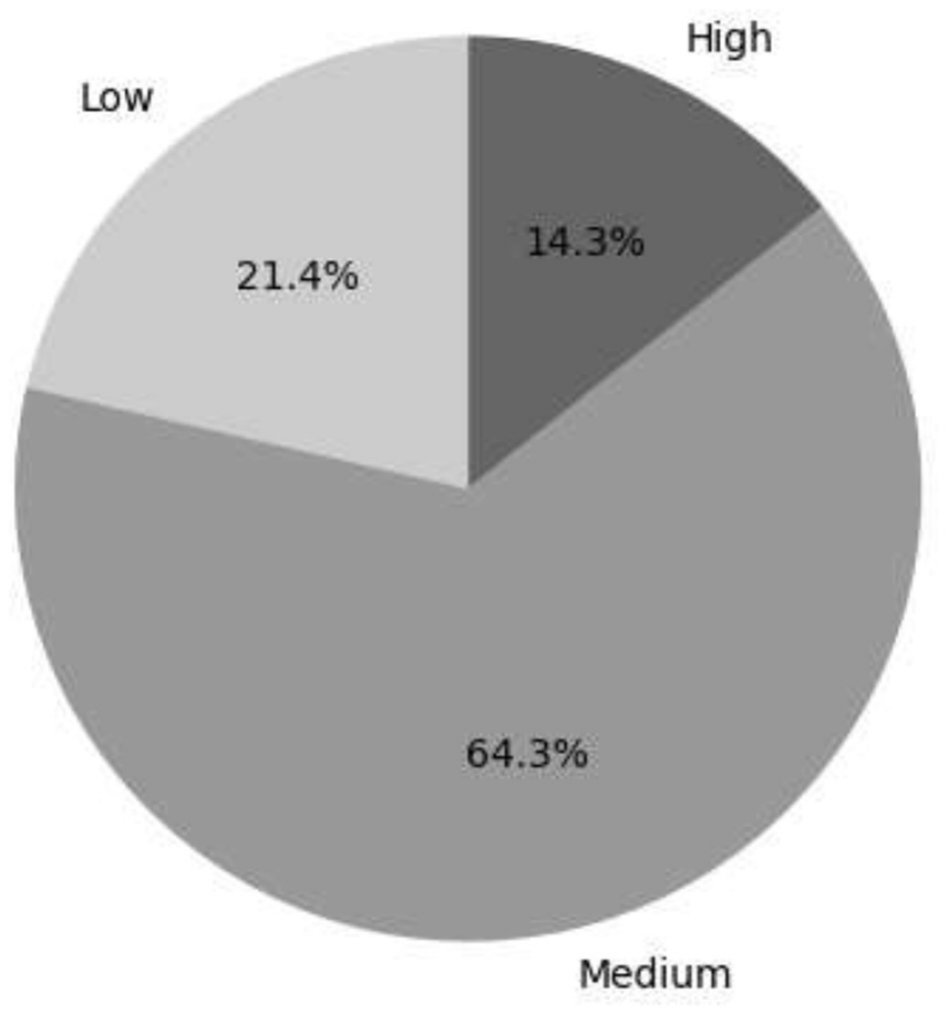
Level of female entrepreneurship.

The descriptive data were analyzed in two variables, for which a three-level assessment level was taken into account for each of the main variables. Figure 4 shows that 14.3% of women entrepreneurs are at a high level, meaning they have access to family support, entrepreneurial skills, support networks, and resources. On the other hand, 21.4% are at a low level, meaning they lack the aspects above. The other part (64.3%) is at the middle level.

**Figure 5:**
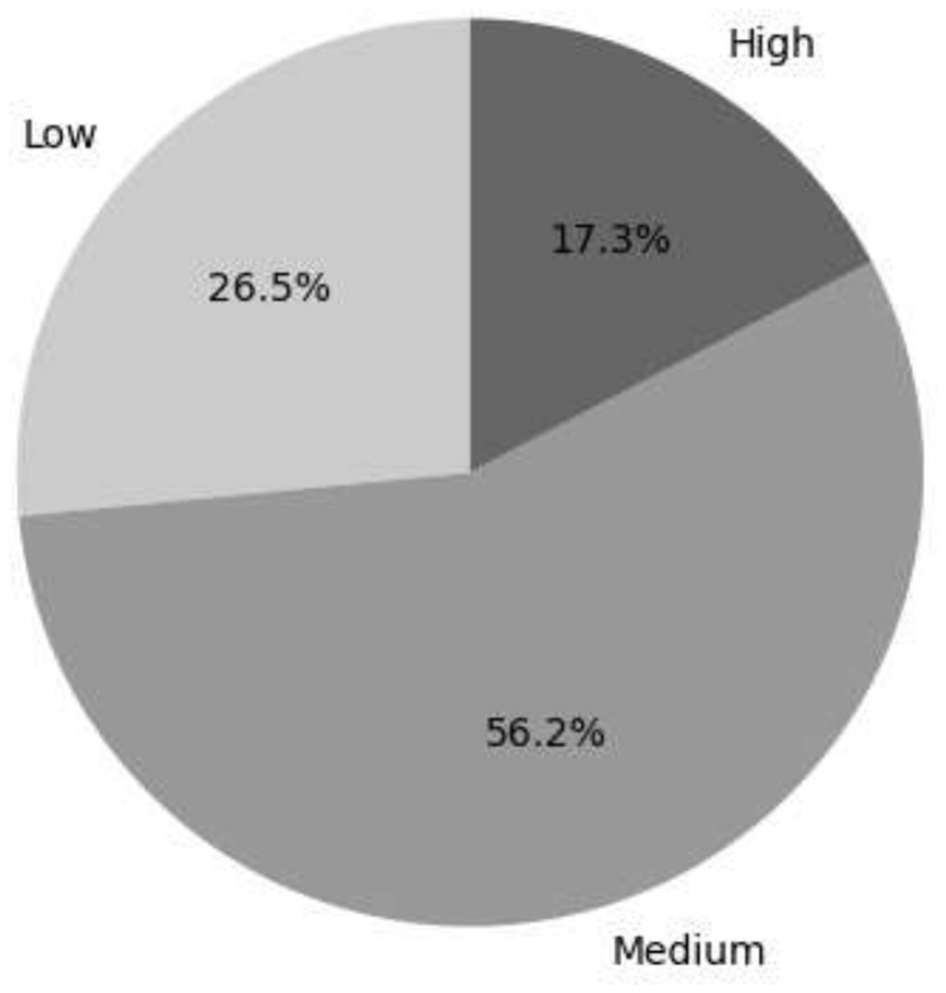
The level of socioeconomic factors in female entrepreneurs.

Figure 5 shows that 17.3% of the female entrepreneurs surveyed consider socioeconomic factors to be highly important, meaning they are important for financial support, connections, and financial independence. However, 26.5% consider them to be less important, while 56.1% consider them to be at a medium level.

#### 4.2.3 Use of structural equations

Partial least squares structural equation modeling (PLS-SEM) was used to analyze the data using SmartPLS 4 software. One of the functions of PLS-SEM is the prediction of the target variable (Mutambara and Bayaga, 2020) in this case, to determine the factors associated with female entrepreneurship.

#### 4.2.4 Model evaluation

The study is composed of a complex research framework because it includes reflexive elements, manifest variables, and latent factors, all of which focus on the same topic of female entrepreneurship. Therefore, using PLS-SEM, the research framework was analyzed in two phases, as suggested by the authors of Hair et al. (2017) and Chin (1998), and the SmartPLS tool was used, which is easy to use, simple, and stands the test of time (Hair et al., 2022). In the first step of the process, the measurement model was analyzed to determine its validity and reliability. The structural model was evaluated for its usefulness in testing hypotheses during the second stage. The reliability of the measurement model was ensured using Cronbach's alpha and composite reliability (CR). The validity of the model was assessed using convergent and discriminant validity tests. Before testing the measurement model hypothesis, the validity and reliability of the measurement model had already been demonstrated. Before using SmartPLS4 software, skewness and kurtosis were applied to assess the normality of the study. Relationships within the structural model were evaluated by testing the significance of the relationships, the explained variance of endogenous variables, and the predictive power of different variables (Sánchez-Prieto et al., 2019).

The study calculates the Variance Inflation Factor (VIF) for each indicator as a measure of internal collinearity, a fundamental step in PLS-SEM models to verify that the predictor variables do not introduce excessive redundancy that distorts the coefficients of the structural paths. The methodological criteria of Hair and Alamer (2022); and Kock (2015) were followed, considering that values below 5 indicate the absence of multicollinearity problems and that values below 3.3 also support the absence of common variance in the method, constituting an appropriate alternative to Harman's single factor test in PLS-SEM contexts.

The external model describes the association between items and latent variables. It is necessary to assess the convergent and discriminant validity of the external model (Hair et al., 2019, 2021b; Salloum et al., 2019) to determine their good fit. Convergent validity assesses the degree of high correlation between theoretically identical latent variables, while discriminant validity assesses the degree of difference between one construct and others (Hair et al., 2021b).

In partial least squares structural equation modeling (PLS-SEM), it is critical to ensure that the indicators represent the constructs to which they belong (Hair et al., 2019). Therefore, various authors agree that those variables that do not reach certain minimum levels of factor loading should be eliminated. Hair et al. (2022) point out that if an indicator has a loading less than 0.40, it should be eliminated immediately, while those with values between 0.40 and 0.70 can be considered for exclusion if doing so improves the reliability of the construct or the average variance extracted (AVE). Along the same lines, Henseler et al. (2009) explain that a loading greater than 0.70 is desirable since it indicates that more than 50% of the variance of the indicator is explained by the construct. Chin (1998) also highlights the importance of maintaining indicators with high loadings, ideally above 0.70, to ensure measurement quality. These criteria allow for refining the model, ensuring that each observed variable contributes meaningfully to the theoretical construct it seeks to represent.

In this sense, when analyzing the external loads (see Table 2), items 14 (0.557) and 15 (0.542) were eliminated from the ES variable; item 19 (0.350) from the AR variable; items 31 (0.600), 32 (0.634) and 33 (0.672) from the FS variable; items 4 (0.599), 5 (0.408) and 6 (0.528) from the IF variable.

**Table 2:** External loads.

**items**	**AR**	**CAP**	**EG**	**FE**	**SE**	**SES**	**SF**	**FI**
FE				1,000				
SES						1,000		
Items 10			0.816					
Items 11			0.882					
Items 12			0.863					
Items 13					0.728			
Items 14					0.557			
Items 15					0.542			
Items 16					0.786			
Items 17					0.838			
Items 18					0.805			
Items 19	0.350							
Items 2								0.744
Items 20	0.753							
Items 21	0.847							
Items 22	0.859							
Items 23	0.857							
Items 24	0.833							
Items 25		0.747						
Items 26		0.820						
Items 27		0.837						
Items 28		0.736						
Items 29		0.829						
Items 3								0.746
Items 30		0.813						
Items 31							0.600	
Items 32							0.634	
Items 33							0.672	
Items 34							0.779	
Items 35							0.838	
Items 36							0.791	
Items 4								0.599
Items 5								0.408
Items 6								0.528
Items 7			0.738					
Items 8			0.834					
Items 9			0.853					
Items 1								0.733

SES, Socioeconomic Factors; FE, Female Entrepreneurship; FI, Household Income; EG, Economic Growth; SE, Social Empowerment; AR, Access to Resources; CAP, Entrepreneurial Capacities; SF, Sociocultural Factors.

## 5 Results

The results were consistent with expectations and support the validity of the proposed model. Several key aspects were analyzed, ranging from the reliability and validity of the constructs to the significance of the structural relationships between the latent variables (Khan et al., 2023; Mutambara and Bayaga, 2020; Neuman, 1994). The reliability and construct validity results were adequate (Table 3), with all constructs showing high internal consistency. Cronbach's alpha coefficients ranged from 0.8 to 0.9, exceeding the minimum threshold of 0.70 recommended by Hair et al. (2020). Similarly, the composite reliabilities (rho_a and rho_c) were greater than 0.88 in all cases, confirming adequate composite reliability according to the criteria of Hair et al. (2020) and Hair et al. (2021a). Likewise, the average variance extracted (AVE) was greater than 0.60 in all constructs, indicating strong convergent validity (Fornell and Larcker, 1981). This suggests that each set of items satisfactorily explains the variance of its respective construct, supporting the factorial structure of the model (Bagozzi, 1978).

**Table 3:** Reliability and construct validity.

**Construct**	**Cronbach's alpha**	**Composite reliability (rho_a)**	**Composite reliability (rho_c)**	**Average Variance Extracted (AVE)**
SF	0.909	0.911	0.943	0.845
EG	0.911	0.914	0.931	0.693
AR	0.890	0.893	0.919	0.695
SE	0.880	0.882	0.918	0.738
CAP	0.885	0.888	0.913	0.636
FI	0.825	0.833	0.895	0.740

Table 4 presents the values of the Heterotrait-Monotrait (HTMT) ratio, used to assess the discriminant validity between the constructs. According to Henseler et al. (2015), HTMT values must be less than 0.90. All coefficients presented meet this criterion, indicating that the constructs are empirically distinct from each other. This evidence supports the discriminant validity of the model, ensuring that each variable measures different concepts, which is essential to avoid theoretical and empirical overlaps.

**Table 4:** Discriminant validity – Heterotrait-Monotrait ratio matrix (HTMT).

**Construct**	**AR**	**CAP**	**EG**	**FE**	**SE**	**SES**	**SF**	**FI**
AR								
CAP	0.616							
EG	0.383	0.378						
FE	0.760	0.852	0.273					
SE	0.119	0.344	0.367	0.122				
SES	0.312	0.376	0.774	0.338	0.547			
SF	0.595	0.734	0.137	0.729	0.229	0.221		
FI	0.118	0.289	0.276	0.298	0.703	0.568	0.301	

Table 5 further strengthens the discriminant validity by the criterion of Fornell and Larcker (1981), where the square root of the AVE (diagonal values) is expected to be greater than the correlations between constructs (off-diagonal values). This criterion is met in all cases, confirming that each construct shares more variance with its indicators than with other constructs. Therefore, both the HTMT and Fornell-Larcker evidence corroborate that the model's structure is discriminatively valid.

**Table 5:** Discriminant validity – Fornell-Larcker criterion.

**Construct**	**AR**	**CAP**	**EG**	**FE**	**SE**	**SES**	**SF**	**FI**
AR	0.834							
CAP	0.552	0.798						
EG	0.336	0.345	0.832					
FE	0.719	0.802	0.265	1.000				
SE	−0.025	0.291	0.331	0.115	0.859			
SES	0.296	0.353	0.742	0.338	0.513	1.000		
SF	0.538	0.660	0.123	0.696	0.190	0.211	0.919	
FI	0.001	0.242	0.244	0.266	0.601	0.520	0.257	0.860

The structural model fit indices are presented in Table 6. The SRMR (Standardized Root Mean Square Residual) value was 0.092 for the saturated model and 0.121 for the estimated model. Although ideally, it should be less than 0.08 (Hair et al., 2022), slightly higher values are still acceptable in PLS models if the structural paths are robust. The d_ULS and d_G indices also remain within acceptable ranges, as does the NFI (Normed Fit Index); although moderate (0.657), it indicates a reasonable fit for complex exploratory models. Overall, the indicators suggest that the model presents an acceptable fit for the study (Hair and Sarstedt, 2019).

**Table 6:** Model fit.

**Fit index**	**Saturated model**	**Estimated model**
SRMR	0.092	0.121
d_ULS	3.665	6.367
d_G	1,717	1927
Chi-square	663,486	728,489
NFI	0.688	0.657

Table 7 shows the R^2^ values for the dependent variables of the model. It is observed that the constructs Entrepreneurial Capabilities (CAP) (R^2^ = 0.644), Economic Growth (EG) (0.550), Access to Resources (AR) (0.517), and Sociocultural Factors (SF) (0.485) have a substantial level of explanation. This indicates that the model has a strong predictive capacity for these factors. In contrast, constructs such as Social Empowerment (SE) (0.263), Family Income (FI) (0.270), and Female Entrepreneurship (FE) (0.114) present more modest values, which is expected considering their more distal position in the model. According to Hair et al. (2022), values between 0.25 and 0.50 are considered moderate, and values above 0.50 are substantial (Gong et al., 2018; Khan et al., 2019).

**Table 7:** R-squared.

**Construct**	**R-squared**	**Adjusted R-squared**
AR	0.517	0.511
CAP	0.644	0.639
EG	0.550	0.544
FE	0.114	0.103
SE	0.263	0.254
SF	0.485	0.478
FI	0.270	0.261

Within the measurement model evaluation, internal collinearity was assessed by computing the Variance Inflation Factor (VIF) for each indicator; as reported in Table 8, the values ranged from 1.00 to 4.37, all below the 5 threshold recommended by J. Hair and Alamer (2022), indicating no multicollinearity concerns. In addition, most VIF values were below 3.3; according to Kock (2015), this supports the absence of significant common method variance, serving as an appropriate alternative to Harman's single-factor test, typically used in exploratory factor analysis, within the PLS-SEM context.

**Table 8:** Internal collinearity statistics (VIF).

**Variable / Item**	**VIF**
ESE	1
IF	1
Var13	1.653
Items1	1.788
Items7	1.798
Items2	1.87
Items3	1.903
Items28	2.133
Items30	2.178
Items20	2.179
Items27	2.328
Items10	2.519
Items8	2.551
Items21	2.582
Items29	2.66
Items18	2.728
Items9	2.763
Items16	2.765
Items23	2.768
Items24	2.823
Items35	2.921
Items36	3.057
Items34	3.116
Items22	3.258
Items17	3.359
Items12	3.496
Items11	3.747
Items25	3.795
Items26	4.369

Table 9 presents the Bayesian Information Criterion (BIC) values for each construct included in the structural model. This indicator is used to evaluate the parsimony of the model, that is, its ability to explain the data with the fewest possible parameters. In general, lower BIC values indicate a better fit with less complexity, which favors more efficient and generalizable models (Raftery, 1995; Schwarz, 1978). In the present study, we observed that the constructs of Economic Growth (EG), Entrepreneurial Capabilities (CAP), and Access to Resources (AR) present the most negative BIC values. This pattern suggests that the model fits these variables more efficiently and with less statistical complexity penalty, which reinforces its relevance within the proposed theoretical framework. It is important to highlight that the BIC does not directly assess statistical significance, but rather acts as a useful comparative measure to contrast alternative models or to evaluate the internal economy of the structural model (Burnham and Anderson, 2004). Its usefulness lies in balancing the model's fit with its simplicity, helping to avoid overfitting that could compromise the model's external validity (Kass and Raftery, 1995).

**Table 9:** Model selection criteria.

**Construct**	**BIF (Bayesian Information Criterion)**
AR	−50.428
CAP	−74,764
EG	−56.134
FE	−1.936
SE	−16.709
SF	−45.273
FI	−17.444

Table 10 shows the total effects between the constructs, that is, the sum of direct and indirect effects. Particularly strong relationships are observed between FE and CAP (0.802), as well as between FE and AR (0.719), supporting hypotheses H4 and H5. Also noteworthy is the effect of SES on EG (0.742) and FE (0.338), showing that socioeconomic factors are a key driver of both economic growth and female entrepreneurship (H2 and H7). In sum, the total effects confirm the central role of the SES construct as a structuring exogenous variable and of FE as a mediating node.

**Table 10:** Total effects of latent variables.

**Construct**	**AR**	**CAP**	**EG**	**FE**	**SE**	**SES**	**SF**	**FI**
AR	1.000	0.552	0.336	0.719	−0.025	0.296	0.538	0.001
CAP	0.552	1.000	0.345	0.802	0.291	0.353	0.660	0.242
EG	0.336	0.345	1.000	0.265	0.331	0.742	0.123	0.244
FE	0.719	0.802	0.265	1.000	0.115	0.338	0.696	0.266
SE	−0.025	0.291	0.331	0.115	1.000	0.513	0.190	0.601
SES	0.296	0.353	0.742	0.338	0.513	1.000	0.211	0.520
SF	0.538	0.660	0.123	0.696	0.190	0.211	1.000	0.257
FI	0.001	0.242	0.242	0.266	0.601	0.520	0.257	1.000

**Figure 6:**
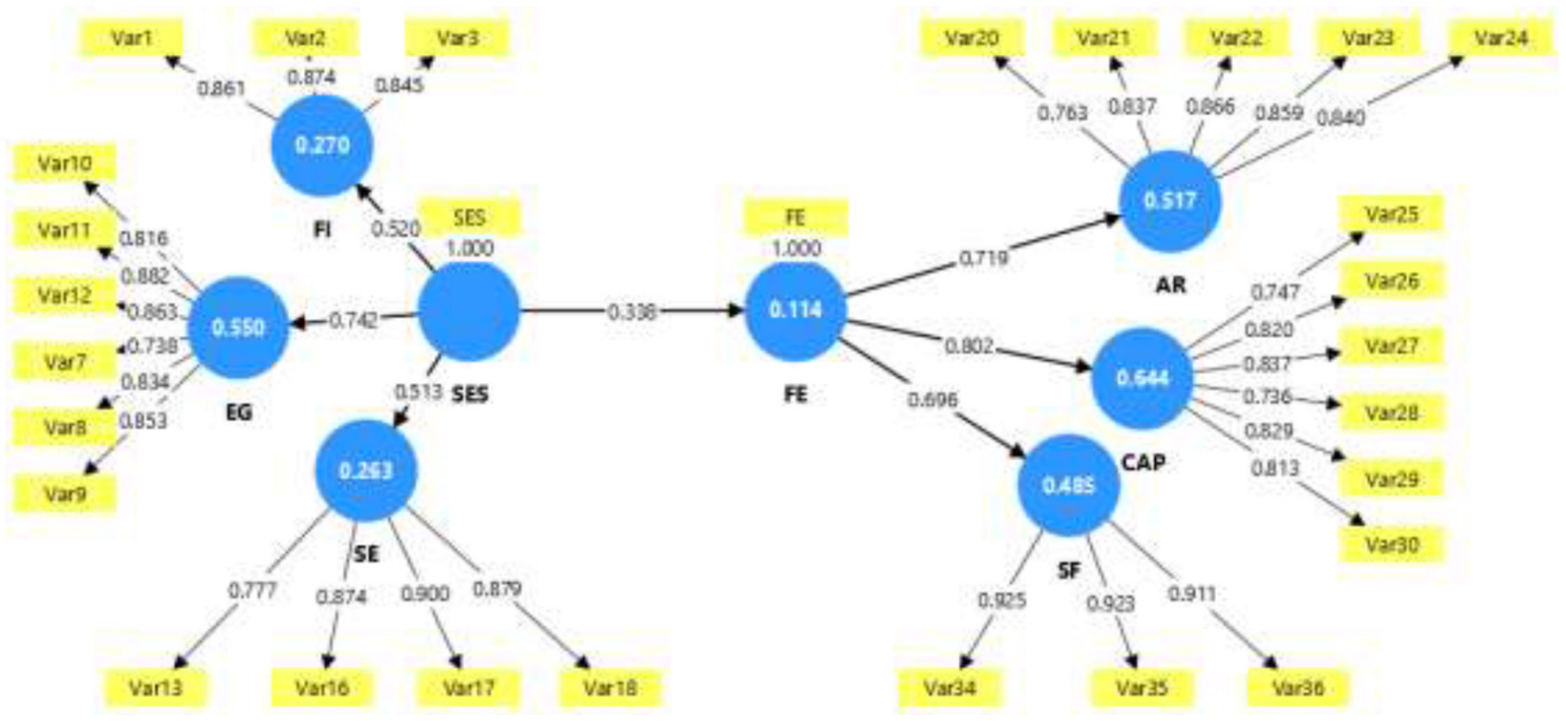
PLS of the model with total effects. SES, Socioeconomic Factors; FE, Female Entrepreneurship; HI, Household Income; EG, Economic Growth; SE, Social Empowerment; AR, Access to Resources; CAP, Entrepreneurial Capacities; SF, Sociocultural Factors.

Table 11 and Figure 6 present the empirical validation of the model hypotheses. All proposed structural paths are significant (*p* < 0.05), with high t values (more than 3.0) and robust path coefficients (between 0.338 and 0.802). Figure 3 provides a clear graphical representation of the structural model, visualizing the direct and significant relationships between the constructs. The arrows indicate directions of influence, and the associated values reflect the magnitude of the overall effects.

**Table 11:** Hypothesis testing.

**Hypothesis**	**Path coefficient**	***f* squared**	**Sample mean**	**Standard deviation**	***t*-statistics**	***p*-values**	**Decision**
FE –> AR	0.719	1,070	0.722	0.053	13.604	0.000	Accept
FE –> CAP	0.802	1,805	0.804	0.040	19,928	0.000	Accept
FE –> SF	0.696	0.940	0.695	0.057	12.284	0.000	Accept
SES –> EG	0.742	1.223	0.741	0.046	16.057	0.000	Accept
SES –> FE	0.338	0.129	0.337	0.112	3.025	0.002	Accept
SES –> SE	0.513	0.385	0.513	0.086	5.947	0.000	Accept
SES –> FI	0.520	0.370	0.523	0.068	7.603	0.000	Accept

The structural model figure confirms that Female Entrepreneurship (FE) is a central node within the dynamics analyzed. The relationship between socioeconomic factors (SES) and female entrepreneurship (coefficient 0.338) shows that the structural environment, educational level, access to services, and household stability, constitutes an enabling foundation for women to undertake entrepreneurial activities; however, the moderate magnitude of this coefficient suggests that structural conditions alone do not explain the decision to become an entrepreneur, as non-structural factors also play a role, such as personal motivations (the pursuit of economic independence, improved quality of life, or social impact), informal support networks (family, community, or peer networks providing financing, knowledge, and emotional support), and individual resilience (the ability to adapt to economic instability, overcome discrimination, and diversify livelihood strategies). These elements complement the structural context and allow female entrepreneurship, once activated, to become a process that not only improves access to resources, strengthens entrepreneurial capabilities, and transforms sociocultural factors, but also sustains and expands itself through these motivations, networks, and resilience, thus positioning itself as a key driver of economic and social development in rural and informal contexts.

In particular, female entrepreneurship strongly influences entrepreneurial capabilities (CAP) with a coefficient of 0.802 and Access to Resources (AR) with 0.719. This shows that the act of entrepreneurship not only involves starting an economic activity but also strengthening the skills, autonomy, and access to the material means necessary to sustain said venture (Hair et al., 2022). A significant relationship is also observed with Sociocultural Factors (SF), which suggests that entrepreneurship can transform beliefs, roles, and cultural patterns associated with women's participation in the economy.

Furthermore, the figure shows how socioeconomic factors directly impact variables such as economic growth (EG, with a coefficient of 0.742) and household income (FI, 0.520). This reveals a dual role of the structural environment: on the one hand, it determines the material conditions for women to become entrepreneurs; on the other, it directly influences household economic outcomes, demonstrating that economic development is intertwined with opportunities for inclusion and equity.

Overall, the model represented in the figure visualizes a coherent and well-integrated structure: structural factors allow female entrepreneurship to emerge, which in turn serves as a bridge to economic, personal, and social development. This interpretation not only validates the architecture of the proposed model but also reinforces the importance of promoting policies that simultaneously address the structural determinants and individual capabilities of women entrepreneurs (Hair et al., 2022; Sarstedt et al., 2017).

The analysis of Table 10 shows that all the hypotheses proposed were empirically validated, given that the path coefficients (β) are statistically significant (*p* < 0.05) and the t values far exceed the critical threshold of 1.96. This indicates that the theoretical relationships between the constructs of the model are supported by empirical evidence. In addition to verifying the statistical significance of the structural relationships, the size of the local effect was evaluated using the f-squared statistic (ƒ^2^), which allows for identifying the degree of impact that an exogenous construct has on an endogenous one in the structural equation model. According to Cohen (2013), *f* ^2^ values are interpreted as (0.02 representing a small effect, 0.15 a medium effect, and 0.35 or more a large effect).

These results show that Female Entrepreneurship (FE) has a very significant impact on Access to Resources (AR), entrepreneurial capabilities (CAP), and Sociocultural Factors (SF), with *f* ^2^ values that far exceed the threshold of 0.35. This suggests that female entrepreneurship acts as a key transformative axis in the socioeconomic structure, enhancing resources, skills, and cultural patterns in vulnerable contexts, as suggested by authors such as Terjesen et al. (2016) and Brush et al. (2019).

Likewise, it is evident that socioeconomic factors (SES) exert a significant effect on economic growth (EG) (*f* ^2^ = _1.223_), family income (FI) (*f* ^2^ = _0.370_), and social empowerment (SE) (*f* ^2^ = _0.358_). These relationships highlight the importance of structural conditions, such as access to services, education, or working conditions, in promoting local economic development. On the other hand, the SES → FE relationship has a small to moderate effect (*f* ^2^ = _0.129_), indicating that, while there is a structural influence on female entrepreneurship, other personal, institutional, or cultural factors may also be influencing its development (Acs et al., 2015; Brixiová et al., 2020). Together, the *f* ^2^ values complement the assessment of statistical significance, providing a more comprehensive view of the weight and importance of each structural relationship in the proposed model (Hair et al., 2022).

## 6 Discussions

This study contributes to the understanding of female entrepreneurship by confirming, through a PLS-SEM model, that socioeconomic factors play a decisive role both in the decision to become an entrepreneur and in the outcomes derived from said activity. Empirical evidence shows that a favorable structural environment, defined by conditions such as financial stability, access to services, and educational level, has a significant, although not necessarily decisive, influence on female entrepreneurship. This finding allows us to contrast the results with authors such as Dencker et al. (2021), who argue that entrepreneurship in vulnerable contexts responds more to conditions of need than to the presence of opportunities. However, the data from this study reveal that even in areas where structural gaps prevail, women undertake entrepreneurship not only as a means of subsistence but also as a means of economic and social growth, challenging the dichotomous vision of need vs. opportunity.

The significant relationship between socioeconomic factors and variables such as family income (β = 0.520), economic growth (β = 0.742), and social empowerment (β = 0.513) reinforces the position of authors such as Hechavarria et al. (2019) and Acs et al. (2015), who argue that adequate structural conditions are a catalyst for inclusive economic development. However, unlike these studies, this work also highlights that entrepreneurship can act as a transformative lever even in scenarios of structural precariousness, provided that there are support networks and family dynamics that support such activity, as proposed by Dewitt et al. (2023).

The results also provide compelling evidence of the role of female entrepreneurship as a transformative agent. Entrepreneurship was found to enable women to expand their access to resources (β = 0.719), strengthen their business capabilities (β = 0.802), and generate changes in sociocultural factors (β = 0.696). These relationships reinforce the arguments of Bianco et al. (2017) and Brush et al. (2019), who highlight that entrepreneurship not only improves women's economic conditions but also challenges and redefines traditional gender roles. However, unlike studies focused on urban contexts or developed economies (Alexeeva-Alexeev et al., 2025), this work demonstrates that this transformation also occurs in peripheral, rural, and highly informal territories, such as the Amazonas region in Peru. This contextual feature introduces specific dynamics of informal female entrepreneurship, such as the predominance of non-institutional support networks, limited access to formal credit, and diversification strategies, that may influence both the motivations and the outcomes of entrepreneurship (Silupu et al., 2024; Xheneti and Madden, 2025). Incorporating this perspective allows for a more nuanced interpretation of the findings and a broader understanding of the strategies adopted by women entrepreneurs in informal contexts.

An important contrast emerges when comparing the relatively moderate effect of socioeconomic factors on the decision to become an entrepreneur (β = 0.338) with the strong impact observed in variables such as economic growth and empowerment. This result suggests that, while environmental conditions are influential, other non-structural elements influence the motivation and capacity to undertake. It is therefore necessary to examine this relationship in greater depth, particularly by considering variables such as personal motivations, informal networks, and individual resilience. This contrasts with research such as that of Coduras and Autio (2013), which emphasizes the macroeconomic environment as a primary factor. In contrast, the findings of the present study are more in line with sociocultural perspectives (Bullough et al., 2022; Marineau et al., 2022), where the decision to become an entrepreneur also responds to regulatory frameworks, gender perceptions, individual resilience, and informal support networks.

Furthermore, the results invite us to rethink the idea that entrepreneurial skills are a prerequisite for entrepreneurship. This study shows that the act of entrepreneurship itself enhances these skills (β = 0.802), which contradicts the literature that presents them as prerequisites (Agarwal and Agrawal, 2023; Hahn et al., 2020). This implies that development policies should not be limited to offering pre-entrepreneurship training, but should actively support women entrepreneurs throughout the process, understanding that skills are progressively consolidated in practice and through access to tangible and intangible resources.

Finally, from a methodological perspective, this paper demonstrates that the structural equation modeling approach (PLS-SEM) allows for adequate modeling of complex realities with multiple interrelated factors, such as female entrepreneurship. Unlike the linear or unidimensional approaches used in other research (Ascher, 2012; Henry et al., 2017), this technique allows for capturing both direct relationships and mediating effects, confirming that entrepreneurship acts as a node that radiates impacts toward different dimensions: economic, social, and cultural.

Taken together, the findings not only support existing theoretical frameworks on the role of women's entrepreneurship in development but also introduce nuances that enrich their understanding. This study calls for the design of more comprehensive policies that combine structural improvements with strategies aimed at strengthening women's individual and collective agency. This implies recognizing women's entrepreneurship not only as an economic response but as a tool for social transformation with the potential to redefine the economic and cultural fabric in historically excluded territories. At the same time, the rural and informal context of this research introduces specific dynamics, such as the predominance of non-institutional support networks, limited access to formal credit, and diversification strategies, that may have influenced the motivations and outcomes of the entrepreneurs. Likewise, although the use of a non-probabilistic convenience sample was necessary to reach this hard-to-access population, it is important to consider how this strategy may affect the external validity of the findings. Acknowledging these methodological aspects does not weaken the results obtained but provides a stronger framework for interpreting their implications and guiding future research in similar contexts.

## 7 Conclusions

This study examined the influence of socioeconomic factors on female entrepreneurship, using a structural equation model (PLS-SEM) that integrated dimensions such as household income, economic growth, social empowerment, access to resources, entrepreneurial capacities, and sociocultural factors. The proposed model showed that structural environmental factors are crucial both for generating material conditions and for strengthening processes of inclusion and economic leadership for women.

The study's findings reveal that female entrepreneurship not only responds to an economic need but also serves as a dynamic mechanism for personal and social transformation. The results showed that entrepreneurship improves access to productive resources, enhances key skills such as planning, management, and leadership, and brings about significant changes in cultural norms and perceptions associated with the role of women. Furthermore, the structural model demonstrated that a favorable socioeconomic environment can strengthen the conditions for entrepreneurship development, increase family income, and contribute to local economic growth.

This work provides a useful empirical framework for understanding how female entrepreneurship can be promoted from a systemic perspective. Similar to digital platforms in the context of industrial traceability, female entrepreneurship acts here as a node that redistributes capabilities, opportunities, and social recognition. Similarly, when a woman becomes an entrepreneur in a structurally strengthened environment, it is possible to trace a series of cascading effects that impact not only her economy but also that of her family, community, and immediate cultural environment.

Likewise, the results help outline concrete actions for institutions, governments, and actors in the entrepreneurial ecosystem to strengthen the conditions necessary for more equitable and sustainable female participation. The design of continuing education and training programs tailored to the reality of rural women entrepreneurs, the improvement of access to microcredit and flexible financial instruments that recognize informality and activity diversification, as well as the promotion of mentoring networks and technical support that consolidate skills and contacts throughout the entrepreneurial process, emerge as key elements for turning female entrepreneurship into a sustainable lever for local development.

In the same way, the findings provide a renewed theoretical framework that positions female entrepreneurship as a transformative agent in rural and informal contexts. This study expands established approaches, such as institutional theory, by showing how formal limitations can be compensated through informal networks, the capability approach by confirming that skills are progressively built in practice, and gender theory in entrepreneurship by documenting changes in norms and cultural perceptions about women's roles. This articulation places the research within the contemporary academic discourse and opens the door to new lines of inquiry that delve deeper into the mechanisms of agency and resilience in highly informal settings.

While the findings of this work provide a significant framework, it is essential to recognize some limitations to contextualize its scope. The sample was non-probabilistic and concentrated in a single territory, which restricts the generalizability of the results. In addition, data were collected through face-to-face surveys at the workplaces and meeting spaces of the female entrepreneurs; although this approach improved the quality and understanding of responses, it may have introduced selection bias by relying on participants' availability. Institutional variables and the analysis of specific public policies were also not included. These limitations do not invalidate the results but delimit their applicability and open avenues for future comparative research across regions, using probabilistic sampling and integrating new dimensions such as digital access, mentoring networks, or government programs.

## Data Availability

The original contributions presented in the study are included in the article/Supplementary material, further inquiries can be directed to the corresponding author.
