# Supplementary material for: Influence of socioeconomic factors on female entrepreneurship: an analysis using structural equation modeling (PLS-SEM)
Source: Front Sociol. 2025 Oct 17;10:1684697. doi: 10.3389/fsoc.2025.1684697 (PMC12576117; doi:10.3389/fsoc.2025.1684697)
Supplement: Supplementary file 1 [file Supplementary_file_1.docx]

**Data Collection Instrument**

| District: ____________ | Age when started the business: __________ | Marital status: _________ |
| --- | --- | --- |
| Product/Service offered: _________ | Is your business formal? Yes ( ) No ( ) | |

Instructions: Please mark one option for each item.
1 = Never, 2 = Rarely, 3 = Sometimes, 4 = Almost Always, 5 = Always

# Variable 1: Socioeconomic Effects

| **Item** | **Question** | **1** | **2** | **3** | **4** | **5** |
| --- | --- | --- | --- | --- | --- | --- |
| **Family Income** | |  |  |  |  |  |
| 1 | How often has your business income significantly improved your household economy? |  |  |  |  |  |
| 2 | How often has your business income covered basic expenses such as food, housing, and education? |  |  |  |  |  |
| 3 | How often has your business income allowed you to save or invest for the future? |  |  |  |  |  |
| 4 | How often have you had access to loans or credit to grow your business? |  |  |  |  |  |
| 5 | How often have you received financial support from relatives or friends to start or expand your business? |  |  |  |  |  |
| 6 | How often have you participated in training programs or financial counseling for entrepreneurs? |  |  |  |  |  |
| **Economic Growth** | |  |  |  |  |  |
| 7 | How often has your business generated employment for other people in your community? |  |  |  |  |  |
| 8 | How often has your business contributed to diversifying the local economy? |  |  |  |  |  |
| 9 | How often has your business driven the creation of new companies in your sector? |  |  |  |  |  |
| 10 | How often do you think your business has contributed to the Gross Domestic Product (GDP) of your region? |  |  |  |  |  |
| 11 | How often has your business generated new products or services that have boosted the local economy? |  |  |  |  |  |
| 12 | How often has your business strengthened local value chains? |  |  |  |  |  |
| **Social Empowerment** | |  |  |  |  |  |
| 13 | How often do you participate in household decision-making related to managing family income? |  |  |  |  |  |
| 14 | How often do you feel heard and valued in decisions that affect your community? |  |  |  |  |  |
| 15 | How often have you assumed leadership roles in community organizations or business associations? |  |  |  |  |  |
| 16 | How often do you feel confident in your ability to generate your own income and maintain financial independence? |  |  |  |  |  |
| 17 | How often do you think entrepreneurship has given you more control over your life and decisions? |  |  |  |  |  |
| 18 | How often do you feel empowered to achieve your professional and personal goals? |  |  |  |  |  |

# Variable 2: Women Entrepreneurship

| **Item** | **Question** | **1** | **2** | **3** | **4** | **5** |
| --- | --- | --- | --- | --- | --- | --- |
| **Access to Resources** | |  |  |  |  |  |
| 1 | How often have you had access to loans or credit to start or expand your business? |  |  |  |  |  |
| 2 | How often have you received financial support from relatives or friends for your business? |  |  |  |  |  |
| 3 | How often have you participated in government or private funding programs for women entrepreneurs? |  |  |  |  |  |
| 4 | How often have you received support from other women entrepreneurs to solve problems or share experiences? |  |  |  |  |  |
| 5 | How often have you participated in networking groups or business associations? |  |  |  |  |  |
| 6 | How often have you received business advice from professionals or mentors? |  |  |  |  |  |
| **Entrepreneurial Skills** | |  |  |  |  |  |
| 7 | Do you consider your educational level fundamental for your business success? |  |  |  |  |  |
| 8 | How often have you applied your academic knowledge to solve problems in your business? |  |  |  |  |  |
| 9 | How often have you felt the need to complement your academic training with specific courses or workshops for entrepreneurs? |  |  |  |  |  |
| 10 | How often have you worked in other businesses before starting your own? |  |  |  |  |  |
| 11 | How often have you used experience gained in other jobs to manage your current business? |  |  |  |  |  |
| 12 | How often have you had to learn new skills to run your business? |  |  |  |  |  |
| **Sociocultural Factors** | |  |  |  |  |  |
| 13 | How often have you faced cultural barriers that hindered your business development? |  |  |  |  |  |
| 14 | How often have you felt discriminated against for being a woman in the business environment? |  |  |  |  |  |
| 15 | How often have you had to adapt your business practices to conform to cultural norms in your environment? |  |  |  |  |  |
| 16 | How often have you received support from your family to start and maintain your business? |  |  |  |  |  |
| 17 | How often has your family shared household tasks to allow you more time for your business? |  |  |  |  |  |
| 18 | How often have you received advice or suggestions from your family to improve your business? |  |  |  |  |  |
